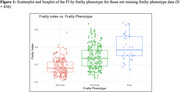# How Sex, Frailty, and Neuropathology Modify the Relationship Between Antidepressant Exposure and Alzheimer's Disease: A Retrospective Study

**DOI:** 10.1002/alz70860_106470

**Published:** 2025-12-23

**Authors:** Lucy Y Eum, Pilar Robinson Gonzalez, Sanja Stanojevic, Melissa K. Andrew, Shanna Claire Trenaman

**Affiliations:** ^1^ Dalhousie University, Halifax, NS, Canada; ^2^ Canadian Consortium on Neurodegeneration in Aging, Halifax, NS, Canada; ^3^ Nova Scotia Health, Halifax, NS, Canada; ^4^ Geriatric Medicine Research Unit, Halifax, NS, Canada

## Abstract

**Background:**

Depression is a modifiable dementia risk factor often treated with antidepressants, but the long‐term association between antidepressant use and Alzheimer's Disease (AD) remains unclear. This study examines the association between antidepressant exposure and AD and, whether binary sex, frailty, or neuropathology modifies this relationship.

**Methods:**

This was a retrospective study using secondary data from a multi‐site cohort study. We analyzed data from 930 participants aged ≥55 with normal cognition. Frailty was measured using a 30‐item frailty index (FI) and frailty phenotype. The primary outcome was the association between antidepressant exposure and clinical diagnosis of AD. Secondary outcomes included potential modification of the association between antidepressant exposure and AD by sex, frailty, or neuropathology index (NPI) scores. The 10‐item NPI was constructed using postmortem AD‐related brain autopsy findings. Logistic regression was used for statistical analysis.

**Results:**

Antidepressant exposure was significantly associated with increased odds of AD (OR 2.51, 95%CI: 1.89‐3.34), and this remained significant when adjusted by the age at baseline, sex, FI, and NPI (OR 3.11, 95%CI: 2.23‐4.37). When stratified by binary sex, antidepressant use was significantly associated with increased AD odds in females only (OR for females 2.87, 95%CI: 2.05‐4.03; OR for males 1.59, 95%CI: 0.91‐2.76), and, after adjusting for the age at baseline, FI, and NPI, the association remained significant for females (OR: 3.93, 95%CI: 2.63‐5.95) and non‐significant for males (OR: 1.86, 95%CI: 1.00‐3.47). For secondary outcomes, the association between antidepressant use and AD was not significantly modified by sex, FI, frailty phenotype, or NPI (*p* >0.05). A scatterplot of FI vs. frailty phenotype showed a positive association between the FI and the frailty phenotype among the subjects not missing frailty phenotype data (Figure 1).

**Conclusion:**

Antidepressant use was significantly associated with increased odds of AD. When stratified by binary sex, antidepressant use was significantly associated with increased AD odds in females, but not in males. Antidepressant‐AD association was not modified by sex, frailty, or NPI. There was a positive association between the FI and the frailty phenotype among those not missing frailty phenotype data. These findings highlight key insights for identifying more modifiable risk factors for dementia.